# A Novel Block Chain Method for Urban Digitization Governance in Birth Registration Field: A Case Study

**DOI:** 10.3390/ijerph19159309

**Published:** 2022-07-29

**Authors:** Jihui Shi, Solomon Kwadwo Nyedu Danquah, Wanhao Dong

**Affiliations:** 1 Department of Management Science, Huzhou University, Huzhou 313000, China; sjh@zjhu.edu.cn; 2 School of Information Engineering, Huzhou University, Huzhou 313000, China; solodanq@gmail.com; 3 School of Public Finance & Administration, Shanghai Lixin University of Accounting and Finance, Shanghai 201209, China

**Keywords:** civil registration, block chain, digitization governance, smart contract

## Abstract

Even though digitization is widely recognized as one of the most imperative trends in achieving effective urban governance, digital infrastructure remains far from the global trend in many African countries. This paper proposes a novel, resilient data manipulation architecture model called the Birth Notification Verification Model (BNVM) using blockchain and smart contracts. The proposed solution was evaluated in a real-world use case scenario in Ghana. The model, which is based on the Ten Civil Registration and Vital Statistics (CRVS) Framework, focuses on the initial inputs for birth registration at the birth notification level. The approach presented in this study paves the way for the creation of decentralized, secure, transparent, and automated systems for civil registration. The application of a smart contract architecture that blends a centralized design with an on-chain and off-chain architecture is further supported by this, providing more evidence of its viability. It offers a safe verification framework for the Ghana Birth and Death Registry based on smart contract technology and can guarantee a birth notification as proof of birth certificate registration in accordance with international standards. The findings provide insight into the use of blockchain technology in public registry institutions. Furthermore, exploring its adoption and implementation in Sub-Saharan Africa contributes to the growing field of blockchain technology research and demonstrates how the concept will address long-standing issues with corruption and security in developing countries.

## 1. Introduction

Digitization governance is one of the most significant current discussions in the urban governance field. Especially, with the spreading of Blockchain Technology (BCT), researchers have shown an increased interest in the application of the technology in solving birth registration challenges as a digital infrastructure in developing countries. According to [1], Blockchain technology enables the shared registry concept from distributed systems to be implemented in a variety of application domains, ranging from cryptocurrency to potentially any industrial system requiring decentralized, robust, trusted, and automated decision-making in a multi-stakeholder environment. The characteristics of the technology could lead to improvements in efficiency, transparency and therefore trust [2]. However, along with this growth in blockchain technological innovations, there is increasing concern over data security and the effectiveness of social systems like civil registrations. Many countries in low and middle-income countries are struggling to achieve adequate coverage and quality civil registrations [3]. Around one-third of all births are not registered. This disparity is most apparent and is known as the scandal of invisibility. In many nations, computerization of birth registration systems at all levels has substantially improved the management and maintenance of an effective system, which has helped to alleviate many of the urban governance difficulties involved.

However, there is little advice for urban governance at present as to how to manage existing or emerging technology pressures or guidance on cross-system coordination and interoperability for efficient and effective Civil Registration and Vital Statistics (CRVS) [4]. In their studies, [5] demonstrates that in countries with a continuous commitment to digital governance, substantial improvement in CRVS systems can be made very fast, particularly when modern Information and Communication Technologies (ICT) are used. Nations of this caliber have enormous potential for advancement, but lack proper access to modern technology, mostly due to a lack of infrastructure. Fundamentally, these nations want transparency, security, and accountability in their operations, all of which are cornerstones of BCT [6].

This study provides an exciting opportunity to make an important contribution to the growing area of urban digitization governance research by exploring the BCT adoption and implementation from the perspective of Sub-Sahara African challenges. The study further evaluates the extent and application of smart contract implementation in a real use case scenario. A blockchain-based system would lead to credible social identity, prevent identity fraud and ease the registration processes. From a broader perspective, robust civil registration and vital statistics systems will propel pursuits towards the achievement of sustainable governance by providing security, facilitating inclusion and access to social services.

It does not seem too difficult to register births with Ghana’s Birth and Death Registry (BDR) even after many years when only an affidavit and a weighing card are required for registration to be completed. This poses a risk and opens the way for fraud [7]. Cases of unapproved amendments of data at the BDR in Ghana and unapproved fees charged by some officials to provide birth certificates to foreign nationals in Ghana have been reported. It can be assumed that an improvement in the security and integrity of the institution’s information system to ensure process verification would curb the current governance challenges.

The main objective of the research is to develop a design model that implements the concepts of BCT to improve processes in birth registration at the Ghana Birth and Death Registry. Specifically, this study aims at achieving the following:

- To investigate the current birth registration processes at the Ghana BDR.
- To identify key pain points as well as relevant blockchain features that can be implemented.
- To propose a new block-chain based solution to tackle identified pain points.
- To test the application of a smart contract solution in the blockchain architecture.

## 2. Literature Review

### 2.1. Background

#### 2.1.1. Civil Registry in Urban Governance

As a basic infrastructure of Urban Governance, civil registration involves the legal notification and recording of individual vital events, including births and deaths, by the government [8]. The office of the civil registrar maintains the records and registers that contain information about vital events, and issues legal certificates on-demand to entitled claimants. This legal data can be used to support further urban planning and digital governance [9]. Birth certificates establish the identity of a person. They also facilitate access to education, employment, economic resources, social protection systems and services, and legal rights and entitlements [10]. In many countries, computerization at all levels of a registration system has greatly facilitated the managing and maintaining of an effective registration system. This is largely due to the increasing use of relatively inexpensive information and communications technology (ICT) that helps to solve many of the issues involved. The use of ICT can also speed up the compilation and availability of vital statistics [11]. A civil registration system encompasses a range of practices involving many institutions. Activities include notifying and registering events and issuing certificates. The ‘Ten CRVS Milestones’ framework is designed to help CRVS stakeholder’s policymakers, managers and development partners better understand how the systems function as a whole, from end to end, by describing the key processes that must be accomplished in any CRVS system to support better governance [3]. Each milestone represents the output or product of several activities that are logically grouped. It also encapsulates a set of requirements that every CRVS system should fulfil [12]. The systematic application of the CRVS Milestone framework exposes neglected aspects in many CRVS systems such as the importance of the anti-epidemic of COVID-19, where the health sector could play a crucial role [13]

#### 2.1.2. Blockchain Technology

The characteristics of blockchain made it an important technology to solve many urban governance problems. Blockchain is a technology that makes the shared registry concept from distributed systems a reality for several application domains, from the cryptocurrency one to potentially any industrial system requiring decentralized, robust, trusted and automated decision-making in a multi-stakeholder situation [1]. Ensuring the trustworthiness of records is a requirement in a range of different contexts where systems of record provide the critical underlying infrastructure necessary to achieve development objectives. This includes organizations responsible for civil registries of births, deaths and marriages, land registries and repositories of financial transactions. Untrustworthy civil registration entries may mean that citizens are unable to prove their identities as a necessary precondition to accessing social protection benefits, or that opportunities for identity fraud emerge that undermine a country’s immigration policies and national security [14]. The characteristics of blockchain technology could lead to improvements in efficiency, transparency and therefore trust [2]. It is characterized by the following features:

- *Peer-to-PeerNetwork infrastructure*—In a P2P network, there is no centralized server, and each user is a node with server functionality. This layer embodies decentralization and network robustness [15]
- *Cryptography*—Blockchain employs cryptography for authentication, permission enforcement, integrity verification, and other areas. It makes use of a variety of cryptographic techniques including cryptographic one-way hash functions, Merkle trees and public key (private-public key pairs) [16].
- *Consensus Mechanism*—In a blockchain network, a consensus is used to prevent dishonest actors from writing potentially invalid information to the database [17].
- *Timestamp*—The process of ‘trusted timestamping’ is an established approach for claiming that particular digital information existed at a particular ‘point in time’ in the past. It is assumed that the time-stamped information is not changeable by anyone in the future. The digital information can be time-stamped by using secure cryptographic methods [18].
- *Ledger*—The ledger represents a list of bundled (data) transactions in cryptographically linked ‘blocks’. Once the transaction data is verified a ‘block’ will be created. The ‘blocks’ in the chain are groups of transactions posted sequentially to the ledger by using a cryptographic signature—that is, added to the ‘chain’ [2].
- *Validity Rule*—Common set of rules of the network (i.e., what transactions are considered valid, how the ledger gets updated, etc.) [12].

#### 2.1.3. Smart Contracts and Time Stamp-Related Research

Zheng et al. classified significant smart contract applications into six groups based on their generalizations [19]. When the transaction request and verification related criteria and conditions are met, smart contracts allow for the transfer of value. Contracts in the actual world are identical to these contracts. The only difference between them is that they are fully digital, which means that they are comprised of a short programming code that is kept inside a distributed ledger system. For example, The use of BCT in trade finance to prove trade-related documents may minimize loan risk, and smart contracts can govern the execution of inter-organizational processes and openly automate delayed or instalment payments, among other things [20]. Smart contracts offer a wide range of potential applications, spanning from the Internet of Things to the sharing economy, among others.

Deth et al., introduced a free-to-use time-stamping service for online news articles. His proposed system uses secure technologies for saving the results of the time-stamped content for example embedding hashes into the Bitcoin blockchain (which is a cryptographically validated blockchain) [18]. Thus, information is present in a distributed blockchain network, making it impossible to get manipulated. Wouda also in his study, proposes an infrastructure for a blockchain-based application to improve the current way real estate is transacted [2]. In the first place, his proposed model could be deployed as a record-keeping tool (blockchain audit trail). Physical and contractual information, related to a property, is recorded and validated by means of blockchain features during the life cycle of a property (operation phase). Validation of related information is summarized in a framework, which is encrypted (hashed) and stored in the blockchain. Again, Lv et al. also designed and developed a catering safety tracing system to solve catering safety traceability problems [21]. Through blockchain technology, the proposed system can ensure the reliability of traceability information. Using a web application and a hybrid APP, users can manage and query trace information. The SSM (Spring + Spring MVC+ Mybatis) framework which was used in web application development simplifies the development and reduces the deployment difficulty. Furthermore, Elisa et al. proposed an e-government framework that can enforce security and privacy in the public sector by employing blockchain technology [22]. The theoretical and qualitative analysis of security and privacy of the framework showed that cryptography, immutability and the decentralized management and control offered by the blockchain technology can provide the required security and privacy in e-government systems. Their proposed system also has the potential of solving the interoperability issues between government departments which is one of the limitations of existing e-government systems. A growing body of literature has elucidated the enormous benefits that distributed ledger technologies like blockchain bring to the table of technological innovations, such as the high transparency on food traceability, high-level autonomy in school credit bank system etc. It is widely an acceptable phenomenon that it solves several verification challenges and thus can be applied to various domains, including CRVS systems [23]. Furthermore, a more appropriate blockchain design is said to be the hybrid architecture in smart contract implementation [24]. However, it is inadequate in terms of implementation and application in a variety of situations, for instance the CRVS system in Africa. Because of this shortcoming, the conclusion’s generalizability is limited, necessitating its application over a wide range of fields. Little attention has been given to the application of blockchain technology in tackling issues related to civil registration and vital statistics. Even so, much of the research has been restricted to Europe and Asia without sufficient attention to its application in the context of Sub-Saharan countries like Ghana. This indicates a need to investigate and implement a blockchain-based solution to address the present verification challenges that exist in Ghana.

## 3. Methodology

### 3.1. Research Approach

For the first research question, an explorative approach is used to identify the current business process of the institution. This involved the use of documents and previous research detailing the process of the current CRVS System. De Savigny recommends a set of documents required for such enquiry [3]. Based on that, an entire process mapping and modelling exercise are subsequently done to describe and analyze the current processes as indicated in Table 1. The ‘Ten CRVS Milestones’ framework proposed by [3,25] as CRVS international standards and best practices is used as a basis for thematic content analysis. Subsequently, pain points in the process maps are identified. The information gathered was utilized to develop the implementation strategy. Its goal was to close the gaps in the present infrastructure and eliminate the pain points associated with verification. A future blockchain design in areas that could be streamlined to improve the performance of the system is proposed. A prototype is then proposed. A live-data prototype is suitable for this research to visualize the BPMN solution. Live-data prototypes are built with HTML, CSS, and JavaScript most of the time. Additionally, they are built on a Local Test Network to illustrate the designed BPMN solution as well as prove its value and motivate actors to implement the solution. The solution orientation can be evaluated by end-users in an early stage and is expected to lead to the improvement of the final design and reduce the chance of critical changes later in the process. The Ethereum platform was selected to develop the decentralized smart contract application for the following factors: It is now considered to be one of the most advanced blockchains available. A solidity programming language is supported, which allows designers to create stateful smart contracts of any complexity using a Turing–complete language.

### 3.2. Modeling

#### 3.2.1. Thematic Content Analysis

Grant and Booth argue that, a qualitative systematic review is a method for integrating or comparing the findings from qualitative studies [26]. The explorative approach is used to identify the current business process of the institution, the author independently carried out the thematic content analysis. This involved the review of documents and previous research detailing the process of the current CRVS System including [7,27,28,29]:

- Reports from previous comprehensive assessments
- Strategic documents containing vision and mission statements, as well as aims and goals of the CRVS system
- Relevant laws and regulations
- Standard operating procedures and workflow diagrams.

Megel and Heermann explained that secondary qualitative data collected can be analyzed using different theories (e.g., grounded theory, interpretative phenomenological analysis, text interpretation (e.g., thematic coding) etc [30]. All the approaches mentioned here use preconceived categories in the analysis. They are ideographic, means they focus on the individual case without reference to a comparison group. Therefore, from the above-mentioned documents, a thematic content analysis method was employed to analyze the secondary data in juxtaposition to the ‘Ten CRVS Milestones’ framework.

#### 3.2.2. Business Process Model and Notation (BPMN)

Process mapping can be used to describe, compare and visualize an organization, processes, workflows and functionality of a CRVS system [10]. We used BPMN to describe and analyze the business architecture of the current system for pain point identification and solution proposition.

## 4. BNVM Model and BPMN Process

### 4.1. The BNVM Model

**Figure 1:**
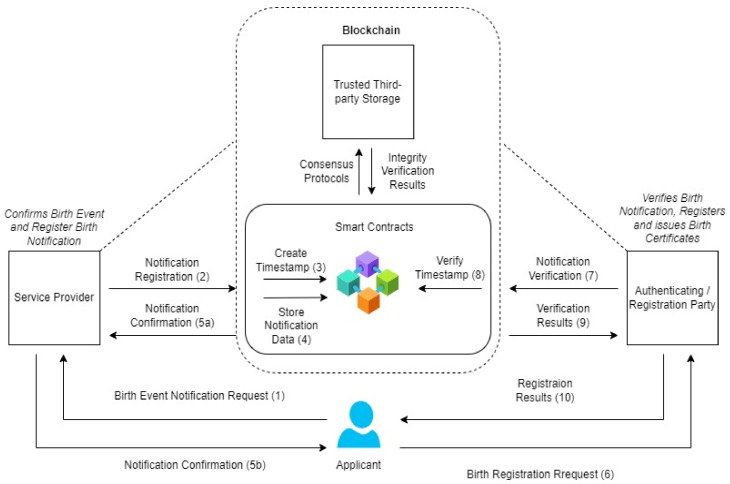
The Birth Notification Verification Model.

Inspired by the Ten CRVS Framework, the model focuses on the initial inputs for birth registration at the birth notification level. A robust architecture that allows birth events to be automatically authenticated prior to birth registration is critical to ensuring a more secured registration system (Figure 1).

Within this model, a birth event is requested by an applicant to the Service Provider (SP) who could be a health professional at a hospital. The service provider registers the notification on the blockchain system that automatically triggers two smart contracts (1) Creation of a Timestamp and (storage of data). After a successful blockchain transaction, the service provider issues a notification confirmation receipt to the applicant for birth registration. Subsequently, a birth registration request is made at the Registration office that represents the authentication or registration party (ARP). They verify the notification receipt on the blockchain with an automatic trigger of the timestamp verification smart contract. After the necessary consensus and verification protocols execution on the blockchain, the verification results are produced for the final internal birth certificate registration processes. Feedback is then given to the applicant. Fundamentally, the blockchain component serves as the verification architecture for the Authenticating and Registration Parties prior to the actual birth registration. Without a successful authentication of the birth notification, the registration party cannot proceed with the issuance of a birth certificate. The model presupposes a dependent relationship between the blockchain, the registration party’s systems and the service provider system.

The model functionality is given in Table 2 below:

Table 2 depicts the algorithm for verifying data according to the proposed model. Firstly, the birth event is declared to a service provider. Secondly, the event details are inputted into the system. After that, ECSC is triggered to store notification data on the blockchain; TSC is further triggered to generate the trusted timestamp for the event. When the ARP inputs the timestamp ID, TVSC is triggered to verify the timestamp on the blockchain. There is an automatic system update after the successful timestamp verification.

### 4.2. Current Registration BPMN Process

**Figure 2:**
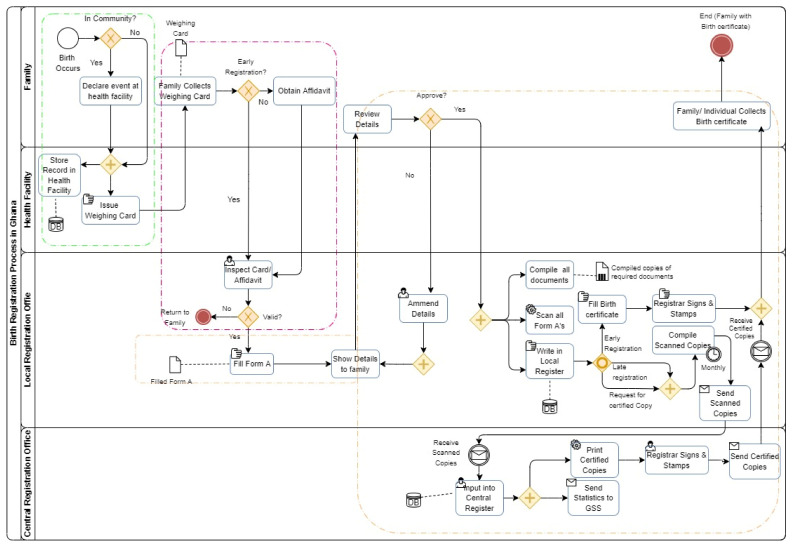
Current Birth Registration Process (BPMN Diagram).

The process is represented visually in the BPMN model. The procedure is divided into three steps: notification, validation/verification, and registration and storage (or archiving). Figure 2 depicts the phases in the process, as well as the activities and stakeholders that are linked with each step in the process.

A parent notifies a healthcare institution of a birth event and, under the current system, obtains a child welfare/weighing card for their kid. When a parent or informant takes their child to the registry to register for new birth, the registration process starts. A child’s welfare or weighing card is required, and the Registration officer fills up Form A when the client provides the necessary information. Because this form is only accessible in English, the registration officer will translate any information submitted to the client into another language. The information on the form is then manually put into a local register, which is subsequently scanned, compiled, and sent to the central register in soft copy. As soon as the birth is recorded for free by the local registrar, the newborn is immediately awarded a birth certificate. The term “late registration” refers to the fact that birth is not documented until the kid reaches the age of 1. Late registrations are handled in the same way as early registrations, with a few exceptions. As with early registrations, the registration process begins at the district level and is overseen by the regional office; however, certificates are only issued in Accra, at the central registry/head office, and are then delivered to the location of registration for collection, unlike early registrations. The process is represented visually in the BPMN model. The procedure is divided into three steps: notification, validation/verification, and registration and storage (or archiving).

#### 4.2.1. Notifications

Table 3 shows the various activities carried out by actors during the notification stage. During the notification phase, the birth event must be declared at a health centre by the family. At the end of the notification process, a weighing card is issued.

#### 4.2.2. Verification/Validation

Table 4 shows the various activities carried out by actors during the verification stage. During the verification and validation phase, the family must collect a welfare or weighing card and an affidavit—if it is late registration. A local registration office would physically inspect these documents and allow you to fill out a Form A registration form.

#### 4.2.3. Registration and Storage

Table 5 shows the various activities carried out by actors in the registration phase. For the time being, families must submit a Form A registration form at a local registration office to proceed to the registration and storage stage. After all, parties have confirmed the information, the local office enters it into a local register, prints up a birth certificate, stamps it, and gives it to the family. Note that this is not a certified copy since it only includes the stamp of the local registrar, not the stamp of the national registrar general. Late registration or certified copy registration are handled by the local registrar who gathers and scans all form A documents once a month and sends them to the central registration office (CRO). A certified copy and a certificate for late registrations are only available from the CRO, which is the only body authorized to issue them. The central register is updated with the scanned information from the local offices, and certified copies are made, signed by the registrar general, and sent to the local offices, from where the family collects them. Following that, handwritten data are sent to the Ghana Statistical Service (GSS).

### 4.3. The Pain Points

The methodology presented above is used to establish and analyze the process’s pain points by comparing them to the Ten CRVS Framework. Using this method, much more clarity and depth may be gained than a general explanation of the process’s pain points. Additionally, by visualizing the present process, it’s simple to understand how the suggested solution fits into it. The framework defines the numerous stages necessary for civil registration. This comparison demonstrates that the whole process is now encountering birth notice and data exchange challenges. The aforementioned pain points are crucial when it comes to confirming and verifying birth events before issuing birth certificates. In the absence of enhanced digitization of birth event notification and data exchange, there is a risk of oversight, as well as unlawful data collection and insertion. Additionally, information processing would be carried out manually, resulting in lengthy delays and inconsistencies throughout the process.

#### 4.3.1. Birth Notifications

**Figure 3:**
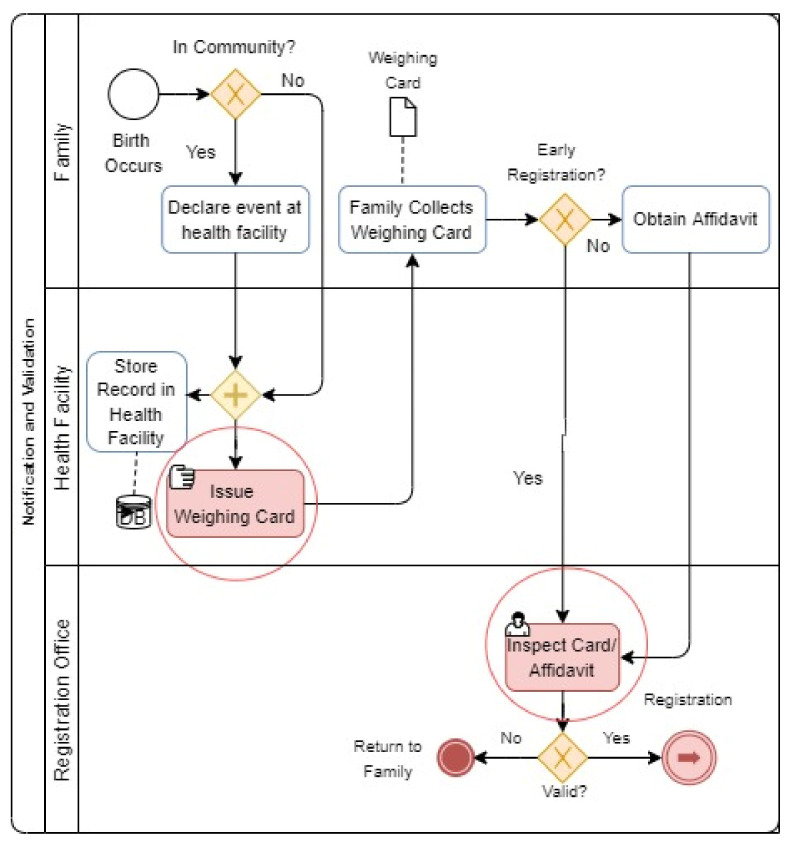
Sharing Pain Point (BPMN Diagram).

As seen in the preceding Figure 3, the system’s persistent issues arise from the notification and verification methods. Among them are the following:

- An undefined method and documentation for birth notification with separate notification information.
- Inadequate integration of notification and registration procedures results in the lack of a centralized record of weighing cards.
- As a result, there are no effective means to validate an individual’s assertion that a weighing card provided as proof for late or delayed birth registration is authentic and correct.
- Illegal modification of weighing card data is neither traceable nor verifiable.

Birth notification, as defined by Cobos Muoz et al. is the collection and subsequent transmission of critical information about the fact of birth or death by a designated agent or official of the CRVS system via a CRVS-authorized notification form (paper or electronic), with the information transmitted being sufficient to support eventual registration and certification of the vital event [31].

#### 4.3.2. Data Sharing

**Figure 4:**
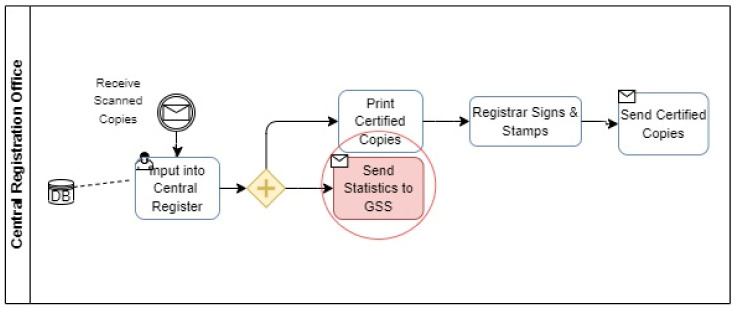
Notification and Verification Pain Points (BPMN Diagram).

Another key problem faced by the system is seen in Figure 4. It was a lack of cooperation with the essential entities for data verification and sharing. Cobos Muoz et al. argues that Coordination across all ministries, agencies and administrative levels is critical to the success of any CRVS system, therefore, plays an extremely vital part in the CRVS Framework [31].

## 5. Proposed Framework

### 5.1. Proposed Registration Process

**Figure 5:**
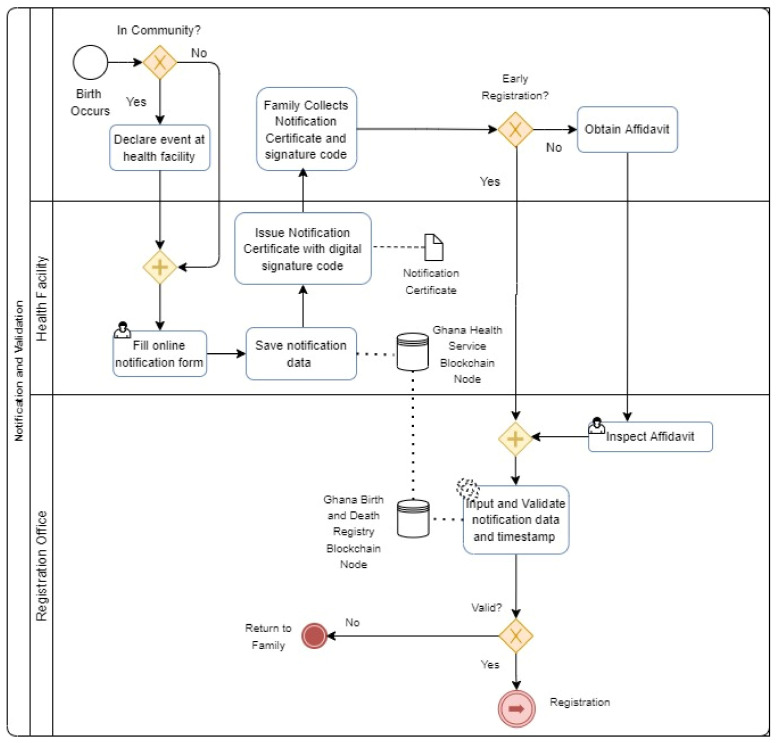
Proposed Notification and Registration Process (BPMN Diagram).

According to the highlighted pain points throughout the registration process, the significant issue was the absence of a digital verification method for the registration system’s main stakeholders, namely the health care workers responsible for birth notification and the BDR Official. As a result, Figure 5 proposes the following workflow utilizing conventional BPMN rules.

#### 5.1.1. Within Each Actor

- Family-The family will be responsible for notifying a health facility of the birth event and obtaining a notification certificate with a signature code instead of a weighing card as documentation of the event for birth registration purposes.
- Health Facility-Certified Health Practitioners will be responsible for completing an online notification form and issuing notification certificates that include all pertinent information about the birth event. Unlike the previous procedure, digital records of the notice will be stored on a central server (GHS node) that acts as a node on a blockchain network.
- Registration Office—In addition to a valid notification certificate (instead of a weighing card), birth registration will need an affidavit (when necessary). The notification data and signature code are entered, which results in automated verification of the data and timestamp, while background investigations are necessary to confirm the validity of an affidavit.

#### 5.1.2. Between Actors

- The approach evaluates two distinct scenarios: at-home delivery and birth in a health centre. Due to the lack of health facilities nationally, several births occur at home and so go unrecognized. In such cases, families must get notification certificates from a health provider within the appropriate birth registration time (usually one year).
- Families are required to attend a local registration centre and provide notification certificates as proof of birth in addition to completing a registration form (Form A). Occasionally, campaigns for birth registration require volunteers from the BDR to travel directly to people’s homes, particularly in remote regions, to register them.
- Local Registration Center and Health Facility- Currently, local registration centres are located in a variety of places, including certain health facilities. In contrast to the existing approach, the BDR will use the blockchain network to share a database among hospitals. This will enable the verification of notification certificates before registration to be automated.

### 5.2. Architecture Overview

**Figure 6:**
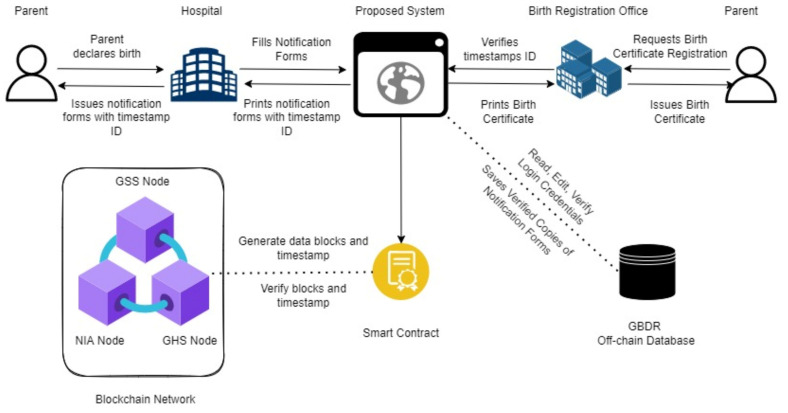
Architecture Overview.

The suggested system is structured in such a way that it addresses the institution’s notification and sharing challenges. As depicted in Figure 6, specific notification forms requesting information by international standards should be used instead of weighing cards as proof of registration notice. The Ghana Health Service, the Ghana Statistical Service, and the National Identification Authority are suggested as nodes on a blockchain network. A smart contract will be launched to store and validate timestamps. A secure timestamp of the date and time of birth event notification registration and subsequent change is critical for maintaining the validity of notification records and hence serves as the birth event verification element. A parent initiates birth notification by declaring a birth event at a hospital or health institution. A licensed health professional accesses the proposed web-based platform, logs in using their practitioner’s license number or login credentials, and completes an online notification form. Once done, the system will generate a timestamp, save a copy of the data off-chain, generate a PDF of the record with a unique timestamp ID, and connect with the smart contract to add the record as a block.

A parent or informant must first seek registration at a local registration facility. A BDR official will be needed to access the site using their given ID/login credentials and complete a birth registration request form that requires just the event notification ID. The smart contract will be invoked in this situation to validate the record and timestamp. After approval, the record is updated to reflect the registration, and a birth certificate may be printed and stamped.

## 6. The Prototype and Evaluation

### 6.1. Design of the Architecture

**Figure 7:**
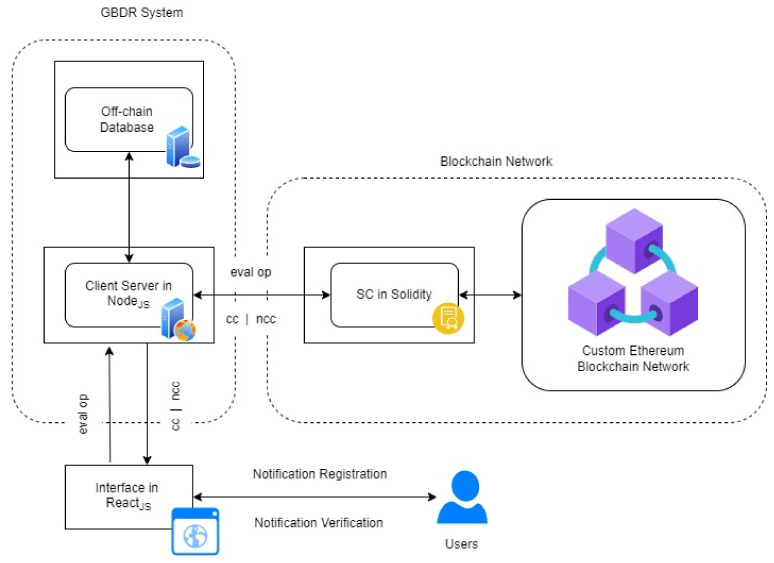
Application Development Components.

Figure 7 illustrates the technologies utilized to build the solution using the Ethereum Blockchain. The contract is deployed using an Ethereum client linked to a Ganache-based private Ethereum network. The Solidity programming language is used to create the blockchain-based smart contract (SC). The Birth Notification System (BNS)—(a Java program)—makes use of the React App client, which serves as a web interface to the NodeJS server. The architecture was used to evaluate the implementation of a smart contract for verification. NodeJS Server communicates with the Ethereum client using the web3j library and Meta Mask.

### 6.2. Smart Contract Deployment

#### 6.2.1. Contract Events

To ensure that the contract executes smoothly, it divides the entire process into two states, corresponding to two events:

- When the HO fills a notification form, it is called to save the information on the blockchain network;
- This is executed when the LRO inputs the timestamp ID and clicks on verify. It is called to authenticate the timestamp ID on the blockchain network;

The contract code snippet above will be executed whenever a registration or verification event is triggered from the front end. This is made possible via the Meta Mask chrome plugin, which links the front end and back end Ethereum networks and provides access to information through the web3.js API.

#### 6.2.2. Timestamp Verification

To further address the shortcomings of the process, we present an approach that automatically generates a publicly verifiable, tamper-proof timestamp for each registration record. This code snippet initiates the off-chain database system to allow the information to be saved. Following this initiation, then call for the execution of the smart contract as the variables within the contract. As long as the time values for the creation of the notification form match, a notification of a Successful Registration Pops Up.

### 6.3. Evaluation and Analysis

To further assess the proposed model, a privacy analysis is carried out on the proposed architecture overview, and a comparative analysis is carried out with the traditional registration process.

#### 6.3.1. Privacy Analysis

Participants in the public Blockchain are free to enter and leave, so the on-chain data are visible to everyone. The privacy of the data cannot be protected. For the model proposed in this paper, consortium Blockchain can be used to authenticate all the participants, and the encryption function of the storage contract and the decryption function of the reading contract can be used to protect the data privacy stored in Blockchain. The multichain structure can protect the privacy of the data. At the same time, no one can view others’ data without permission.

Based on the Novel BNVM, the proposed architecture used to ensure the privacy of patients or applicants and verification of notification records can strengthen the trust between the GBDR and GHS and reduce the running costs of registration and maintain the security of the whole system. It has the following advantages in terms of privacy and security.

- Good fault tolerance. The decentralized system does not depend on a single service node and can effectively solve the single-point failure. Multiple service nodes depend on each other to reduce the possibility of errors. Decentralized, autonomous, and distributed nature will bring spontaneous innovation and new ways to coexist with efficiency.
- Attack protection. The centralized system is easily attacked by a third party or becomes an attacker, while the decentralized system has no centralization. If malicious nodes attack all nodes on the whole system, they will pay a high cost, so the possibility of evil is very low.
- It was preventing monopoly. Compared with the centralized system, it is difficult to use the information asymmetry to collude with each other in the decentralized system. A decentralized system allows all nodes to participate in the decision-making, ensuring the system’s security and transparency. At the same time, it also can audit malicious behaviours.
- They are improving the trust between participants. The decentralized system has no unified third party, so all participants do not have to bear the risk caused by trusting the third party. The system can automatically facilitate cooperation between the two parties.

#### 6.3.2. Compared with the Current System

The proposed architecture combining blockchain and smart contract technology is different from the current system. Table 6 mainly compares this model with the current system from five aspects, including the data integrity of birth notification records, the data storage persistence of notification and registration records, The difficulty of verification, and compatibility. The proposed model provides a good foundation for the transformation of urban digital governance in future.

## 7. Discussion

In light of the blockchain solution deployment lifecycle proposed by [32], this study discusses the solution in three areas: the benefits, deployment and feasibility.

### 7.1. The Benifits

As introduced in Section 5 and illustrated in Figure 5 and Figure 6. The centralized nature of the proposed system has the potential of facilitating direct interactions between public institutions and citizens. Following the evidence by [32], the append-only way of updating the blocks ensures the irrevocability of a ledger and increases the integrity and verifiability of data. It reduces operational risk and transactional costs as well as increases compliance and trust in government institutions. Its security will ensure proper and efficient use of public funds, decrease the cost of human verification and expedite process coordination.

### 7.2. The Deployment

The deployment of smart contract is the key to gurantee the data security and privacy. Core requirements for deployment will involve considerations for technologies like cloud computing as demonstrated by [33,34]. The proposed architecture bridges the gap between the Ghana Health Service and the Ghana Births and Deaths Registry by demonstrating how a BNVM enhances the security and integrity of the information system. However, critical issues relating to data privacy and relevant policies to ensure the usage of the system by the necessary parties are paramount to the successful deployment of the solution. As a government system, data is expected to increase vastly over the years. As a result, an implementation should involve the use of side-chains and demand lightweight data to be stored on-chain for verification only. Laws and international standards for civil registration and vital statistics fully support the critical need for data sharing among the cited nodes [10,35,36]. Currently, the government is deploying a blockchain solution for the management of land registries [37]. Therefore, it can be assumed that the blockchain infrastructure experience would be present for the general deployment of the proposed solution.

### 7.3. The Feasibility

This research provides additional evidence of the applicability of a smart contract architecture that combines an Ethereum-based blockchain platform with a centralized database design [24]. It provides a secure and feasible verification framework for GBDR that is based on blockchain technology and can ensure a birth notification as proof of birth certificate registration per international standards as described by [35].

## 8. Conclusions

### 8.1. Theoretical Contribution

A large and growing body of literature has investigated the broad application of blockchain technology to solve different private and e-government challenges of reliability and traceability of data to ensure security [2,6,18,26]. However, little attention has been given to the tackling of issues related to civil registration and vital statistics. In their studies, Somaiman et al. presented and evaluated the performance of a novel hybrid architecture for the implementation of a smart contract [24]. However, much uncertainty still exists about the applicability of the architecture in solving e-government challenges especially in developing countries. This study introduced a resilient blockchain-based design solution, BNVM, for resolving birth registration verification difficulties to help in transforming the digital governance of Ghana. It provides additional evidence of the applicability of a smart contract architecture that combines an Ethereum-based blockchain platform with a centralized database design.

### 8.2. Practical Contribution

The findings and proposals from this study suggests several courses of action for practitioners. Government database architectures can now move closer to implementing a blockchain based system by offering a safe verification framework for the Ghana Birth and Death Registry based on smart contract technology. Additionally, by connecting civil registration with other government systems like identification and health, it will be possible to improve CRVS quickly and facilitate interoperability across all industries.

### 8.3. Study Limitations

The limitations of this research are inherent in its scope. The goal of this study is to optimise Ghana’s civil registry’s birth registration process. Although the proposed blockchain approach addresses the process’s current pain points and difficulties, the study’s depth is constrained. For instance, the data sources are restricted to published empirical research. An empirical inquiry could be conducted to affirm the validity of the processes discovered. Additionally, research on technical and legal subjects is not included in this study. These issues are left out of scope due to the author’s insufficient knowledge of them. As is the case with all emerging technologies, the majority of research on blockchain technology is focused on designing frameworks.

### 8.4. Suggestions for Further Research

Future researchers could repeat this study by assessing the applicability of the framework in international settings. As the first study to concentrate on civil registry optimization using a blockchain framework, more study is required to test the concept using an application. Additionally, investigations into the legal and technical aspects of implementations could be conducted.

## Figures and Tables

**Table 1:** Research Design.

Research Question	Data Source	Data Analysis	End-Product
What does a birth registration process in Ghana look like?	Documents: Comprehensive assessments reports Standard operating procedures	Thematic Analysis Business Process Mapping and Notation (BPMN)	BPMN Diagram
What pain points occur during birth registration in Ghana?	BPMN Diagram	The Ten (10) CRVS Framework	Proposed BPMN Diagram
What does the proposed blockchain-based solution look like?	Blockchain Literature	Analysis based on the problem statement	Prototype: Solidity Node.js React App MongoDB Ganache

**Table 2:** Explain of BNVM Functionality.

**Functionality:** Using Blockchain and Smart Contract Technology to guarantee data manipulation fraud in BNVM
**Input:** Birth Event Details
**Output:** System Status
**Step 1.** Birth Event is declared to a service provider
**Step 2.** SP records details on the blockchain system
**Step 3.** Event Creation Smart Contract (ECSC) is triggered.
**Step 4.** Timestamp Creation Smart Contract (TCSC) is triggered.
**Step 5.** ARP inputs timestamp ID upon birth registration request by applicant
**Step 6.** Timestamp Verification Smart Contract (TVSC) is triggered.
**Step 7.** Verification results is given.
**Step 8.** A system update necessary for final registration steps and issuance of birth certificates

**Table 3:** Notification Actors and Activities.

Involved Actors	Family, Health Facility
Task in BPMN	Declare Birth Event Issue Weighing Card Store Record

**Table 4:** Verification/Validation Actors and Activities.

Involved Actors	Family, Local Registration Office
Task in BPMN	Obtain a welfare Card, Weighing Card and or an affidavit Inspect Card

**Table 5:** Registration Actors and Activities.

Involved Actors	Family, Local Registration Office, Central Registration Office
Task in BPMN	Fill Form A Show details to family Review Details Amend details if necessary Compile all documents Scan all Form A’s Write in the Local Register Fill out Birth Certificate (Uncertified Copy) Local Registrar Stamps Compile scanned copies Send scanned copies every month Input details in a central register Print certified copies Registrar General Signs and Stamps Send Certified Copies to Family Family Collects Certified Copies at Local Office Send Statistics to GSS

**Table 6:** Proposed architecture compared to the current system.

Index	Current System	Proposed Architecture
**Data integrity**	It is easy to destroy data integrity because of hardware failure, network failure, logic problems, unexpected catastrophic events, and human activity.	It dramatically reduces external interference and human-made damage. The Blockchain participants cannot arbitrarily destroy the integrity of the data because of the several reproductions existence.
**Data storage** **persistence**	The registration data is mainly stored in the server. If the server is damaged, the data is completely lost.	Registration data has multiple backups so data storage has better persistence.
**The difficulty of** **Verification**	It is challenging and complex to verify notification data because the information sources are not easily verifiable, so the verification process is not efficient.	It automatically verifies the existence of a birth event using timestamp smart contract, so it saves manpower and time.
**Compatibility**	Different third-party institutions have different civil registry data requirements. Because of the lack of a sharing system.	The connected blockchain notification system has the potential for further development of dashboard for different nodes to view data in real-time and meaningful ways.
